# A Vade Mecum for New Year's Givers

**Published:** 1890-01-11

**Authors:** 


### A Vade Mecum for New Year's Givers.

(*Continued from last week's* The Hospital, *pp. 218-22.*)

There were a few institutions the aims of which we were unable to advocate for want of information or from other causes last week, and we therefore gladly recur to the subject of New Year's gifts. Any philanthropist who will study the *Vade Mecum*, as it appeared in our last week's issue, and who will read what follows, need have no difficulty in making a wise selection of recipients for his bounty. There is nothing more cheering to those who have to bear the burden of financial responsibility entailed by the efficient maintenance of a great charity, than a liberal contribution at the commencement of a new year. We hope our readers will bear this fact in mind, and that the *Vade Mecum* of 1890 may prove the means of considerably augmenting the available funds of many a deserving institution.

### GENERAL HOSPITALS.

**German Hospital,** Dalston-lane, E. Secretary, Mr. C. Feldman.—This institution is free to Germans and German-speaking people, but accidents and other urgent cases are admitted independently of considerations of nationality. The Duke of Cambridge is the president, and takes a genuine interest in the management and progress of this excellent hospital. It has two branch establishments, one at Aldgate and one at 259, Oxford-street, W., where out-patients are seen and prescribed for. Funds are needed, and will be gratefully acknowledged by the secretary.

**Guy's Hospital** has of late years fallen from the proud position of independence which it so long shared with Bartholomew's. It has been forced to seek for help, and having received all it demanded has apparently lost its self-respect, and goes on, like Oliver Twist, asking for more. There is, however, a famous medical school attached to the hospital, to which a residential college has lately been added, and it may reasonably be hoped that any donations given to it will profit the suffering poor of the large and thickly-populated district in which it is situated.

**St. Bartholomew's Hospital** is one of the few fortunate institutions which need no help from any man. It has, however, a nursing home attached to it, which will benefit largely by donations for increasing its size and adding to the comfort of the nurses belonging to it. There is besides a Samaritan fund used to help discharged patients, for which contributions are as desirable.

**St. Thomas's Hospital** is fortunate in being fully endowed, but as none of the funds thus obtained can be devoted to the assistance of the convalescent patients there is even here room for charity. The Nightingale nurses, who are trained within its walls as a memorial of one of the noblest women of modern times, should, by the good work they do, be the best recommendation of the hospital where they were educated.

### FEVER HOSPITALS.

**London Fever Hospital,** Liverpool-road, N., has fortunately not had such a run upon its accommodation in 1889 as in the preceding year. It must, however, be kept up ready for the possible outbreak of an epidemic, as well as for those occasional cases of infectious disease which are sure to be met with in a large city. The staff of nurses is added to when the number of patients increases, but a considerable number must always be kept ready for the chance of patients appearing, and this causes the amount of annual expenditure to seem large when compared with the number of patients treated. Without such expenditure efficiency would be impossible, and though it is satisfactory to note that patients' fees form nearly a third of the income, there is ample room for donations from those who have suffered from infectious diseases. Though they may not have been treated within its walls, they must remember that the existence of an isolation hospital may often prevent an epidemic from assuming serious proportions. The committee of this institution are called upon to provide daily at this season for close upon 100 patients of all ages and both sexes suffering from scarlet fever, diphtheria, and other forms of infectious disease. The benevolent are earnestly asked to help. Subscriptions and donations sent to Major Christie, the secretary, at the hospital, will be gratefully received and acknowledged.

### HOSPITALS FOR CHILDREN.

**Home and Infirmary for Sick Children,** Lower Sydenham. Hon. Secretary, Mr. William Ast.—Established in 1872, it now contains forty-five beds, of which forty are on an average constantly occupied. About 3,000 out-patients are relieved annually. Each patient if provided with a subscriber's letter pays 2s. 6d., or otherwise 7s. 6d. a week. Patients are only admitted on Mondays and Thursdays. In addition to the beds to which paying patients are admitted, there are also twelve free cots maintained by individual subscriptions.

**Belgrave Hospital for Children,** 77, 79, Gloucester-street, S.W.—This institution, established in 1866, contains twenty-two beds and relieves upwards of 3,000 children every year at an expenditure of less than £1,500. It speaks well for the management, and the interest it is able to command, that not less than ten of the twenty-two beds are endowed by subscription, the cost of such endowment being £30 per annum per cot. In addition to its ordinary work it maintains a Samaritan fund, which is devoted to the alleviation of the sufferings of the children when removed from the hospital, or when the hospital is too full to receive them, during the period of sickness rendered all the more trying by the circumstances of their homes and surroundings. The hon. secretary is Captain W. T. Stopford, and Miss Monro is the lady superintendent, either of whom will be glad to receive contributions.

**Hospital for Sick Children.**—This institution was founded in 1852 in Great Ormond-street, and now contains 127 cots. It also has a convalescent branch at Cromwell House, Highgate, which holds 52 cots. The vast amount of good work done by this hospital entitles it to receive most generous contributions. How disappointing, therefore, to know that the assured income is less than £470, whilst the average annual expenditure exceeds £9,000. Such a record for the first children's hospital in Great Britain demands improvement, and we would urge upon all thoughtful readers the desirability of subscribing annually to this great charity. There are few places where an hour can be more pleasantly, or profitably spent than at the Hospital for Sick Children, especially if Miss Phillipa Hicks, the lady superintendent, or Mr. Adrian Hope, the secretary, be in attendance as cicerone.

### MISCELLANEOUS.

**Surgical Aid Society,** Salisbury-court, Fleet-street E.C. Mr. William Tressider, secretary.—Trusses, elastic stockings, artificial limbs, and every description of surgical instruments are supplied by this useful society to the afflicted poor, without limit as to locality or disease. No doubt the sympathies of the public are more easily aroused by the sight of wards and sick beds, but there is a necessary work in addition to curing those who are actually sick, *i.e.*, relieving sufferers who are to all appearances healthy and well. This is the work of the Surgical Aid Society, and Mr. Tressider has succeeded in guiding its operations effectually.

